# Changes in Risk-Taking Behaviors during the First Year of College in the Northwestern Iran: A Latent Transition Analysis

**Published:** 2019-10-21

**Authors:** Soudabeh Marin, Hamid Allahverdipour, Mohammad Hajizadeh, Ali Fakhari, Hossein Ansari, Asghar Mohammadpoorasl

**Affiliations:** ^1^Department of Statistics and Epidemiology, Faculty of Health, Tabriz University of Medical Sciences, Tabriz, Iran; ^2^Research Center of Psychiatry and Behavioral Sciences, Tabriz University of Medical Sciences, Tabriz, Iran; ^3^School of Health Administration, Faculty of Health, Dalhousie University, Halifax, Canada; ^4^Research Center of Psychiatry and Behavioral Sciences, Tabriz University of Medical Sciences, Tabriz, Iran; ^5^Health Promotion Research Center, Zahedan University of Medical Sciences, Zahedan, Iran; ^6^Research Center of Psychiatry and Behavioral Sciences, Tabriz University of Medical Sciences, Tabriz, Iran

**Keywords:** Health risk behaviors, Tobacco, Smoking, Substance abuse

## Abstract

**Background:** First year of university is a critical life transition period with an increased probability of taking risky behaviors. We aimed to examine the transitions in risky behaviors in the first year of college in the northwestern Iran using latent transition analysis (LTA).

**Study design:** A longitudinal study.

**Methods:** A random sample of 1406 freshmen enrolled in three universities in Tabriz City (the capital city of East Azerbaijan Province, northwestern Iran) were evaluated twice in November 2014 and 2015. A multiple-choice self-administered questionnaire was used to collect data on risky behaviors and demographic characteristics of students. LTA was performed to examine the prevalence and changes in risky behaviors (cigarette and hookah smoking, illicit drug use, alcohol consumption and risky sexual behaviors) among the students.

**Results:** The LTA model revealed four interpretable statuses. The prevalence data showed that 77.1%, 12.3%, 8.3% and 2.3% of students were "risk-free", "tobacco user", "sexual risk-taker" and "multiple risk-tacker", respectively in the first assessment. Over the period of one year, 4.2% and 6.1% of risk-free students became tobacco user and sexual risk-takers, respectively, and 22.4% of tobacco user students, transfer to the multiple risk-taker group.

**Conclusion:** The prevalence of risk-taking behaviors increased during the freshman year. Tobacco smoking was predispose acquiring more risky behaviors. Probability of transition to the multiple risktaker group was higher among tobacco users.

## Introduction

First year of the college, which coincides with emerging adulthood, is a major developmental period. Students experience increased independence, responsibilities and change in their lifestyle^1^. This period is also a critical time for the establishment of health behaviors. The first-year college students are more prone to take risky behaviors^2-5^. For example, a study on hookah smoking behavior among college students^3^ suggested an increased in the use of hookah by 2.1% during the first month of college. Other studies^6, 7^ reported a high prevalence and initiation of smoking and illicit drug use (e.g. marijuana) during the freshman year. A representative study in Iranian medical students showed that approximately 37% engaged in at least one risk behavior{Poorolajal, 2018 #54} ^8^.

Combination and co-occurrence of risky behaviors are other health problems among college students^9-11^, which hinders the prevention programs on risky behaviors. Tavolacci and colleagues reported an increased prevalence of tobacco smoking and cannabis use linked to alcohol consumption among college students in France^12^. A relationship was found between hookah smoking and the prevalence of cigarette smoking, cannabis, cocaine, amphetamine and alcohol use in college students in the United States^13^. Co-occurrence of alcohol consumption and risky sexual behaviors^14^, alcohol consumption and cigarette smoking^15^, illicit drug use and risky sexual behaviors were documented in other studies^8, 16^.

Although the high proportion of young people attending college in Iran, little is known about the prevalence and transition of risky behaviors in Iranian college students. To fill the gap in the current literature, this longitudinal study aimed to examine the prevalence and changes in risky behaviors (cigarette and hookah smoking, illicit drug use, alcohol consumption and risky sexual behaviors) using Latent Transition Analysis (LTA) among first-year college students in Tabriz City – the capital city of East Azerbaijan Province, in northwestern Iran.

## Methods

In this longitudinal study, a representative sample of freshmen undergraduate students (n=1406) in the fall semester of 2014 were selected from the three major universities in Tabriz: Tabriz University, Tabriz University of Medical Sciences and Islamic Azad University of Tabriz. Students were selected randomly based on proportional cluster sampling method. Based on the proportion of freshman students in each university, classrooms were selected randomly and then all students from selected classes were enrolled.

The participants were assured about the confidentiality of the information and voluntary nature of participation in the study. The study and its questionnaire were approved by the Ethics Committee of Tabriz University of Medical Sciences.

Risk-taking behaviors of students were assessed twice, with 12 months apart. A multiple-choice self-administered questionnaire was completed by the participants one in the baseline (November 2014) and another in the follow-up year (November 2015). Respondents were assured about the confidentiality and anonymity of the responses given in the questionnaire. Information about demographics, cigarette smoking status, hookah smoking status, illicit drug use and risky sexual behaviors was obtained by the questionnaire.

Five dichotomous variables were used to assess risk-taking behaviors of the participants. These variables include: a) cigarette smoking (smoking 100 cigarettes or more in lifetime, irrespective of current smoking status), b) alcohol use in the past 30 d, c) hookah use (at least once per month), d) ever substance abuse, and e) having unsafe sex (ever) (i.e., using drugs or alcohol before the last sexual intercourse, having sexual intercourse with several people, or having sex without using condom to prevent sexually transmitted diseases). The inclusion criteria included: freshmen students and willing to participate in the study. The incorrectly filled out questionnaires were excluded from the analysis.

### Statistical Analysis

Five indicator variables of cigarette smoking status, hookah smoking status, illicit drug use, alcohol consumption and involving risky sexual behaviors were used to perform the LTA models. The Likelihood-ratio (G^2^) test, Akaike Information Criterion (AIC) and Bayesian Information Criterion (BIC) were used to compare the relative fit of models with different numbers of latent statuses (a lower AIC or BIC value indicates a better fit). Parsimony and model interpretability were also considered in order to determine the optimal number of latent statuses. The analyses were performed using SAS software, ver.9.2, and procLTA software procedure for LTA.

## Results

Of the 1406 sampled students, 1311 participated and completed the anonymous questionnaire (response rate 93.2%). The participants’ age ranged from 17 to 32 yr (mean, 19.48±2.68 yr). The majority of students in the sample were female (53.2%) and 6.0% were married. After a one-year follow-up, 300 (22.9%) students lost to follow-up from the study. A summary of students’ risky behaviors in two times is presented in Table 1. The prevalence of all the risky behaviors in the baseline (time 1) were generally lower than the follow-up year (time 2).

**Table 1:** Proportion of participants reporting risky behaviors in the baseline (time 1) and follow-up (time 2) years

**Type**	**Time 1 (n= 1311)**		**Time 2 (n= 1011)**	
	**n**	**% Proportion** **(95% CI)**	**n**	**% Proportion** **(95% CI)**
Smoking	92	7.0 (5.8, 8.5)	118	11.7 (9.8, 13.8)
Hookah use	77	5.9 (4.7, 7.3)	76	7.5 (6.1, 9.3)
Alcohol use	33	2.5 (1.8, 3.5)	54	5.3 (4.1, 6.9)
Substance abuse	28	2.1 (1.5, 3.1)	30	3.0 (2.1, 4.2)
Risky sexual behavior	129	10.0 (8.5, 11.7)	164	16.4 (14.1, 18.6)

Considering the 2 time periods and 5 dichotomous risk-taking behaviors indicators 64 response patterns could be observed. LTA models with statuses ranging from 2 to 6 were fitted and for each model, G^2^, AIC and BIC were computed (Table 2). According to the results and interpretability of the results of models, the four latent statuses model was an appropriate model.

**Table 2:** Comparison of LTA models with different latent statuses based on model selection statistics

**Number of latent statuses**	**Number of Parameters estimated**	**G** ^ 2 ^	**df**	**maximum log-likelihood**	**AIC**	**BIC**
2	23	871.3	1000	-2317.3	917.3	1036.6
3	38	677.1	985	-2220.2	753.1	950.1
4	55	458.6	968	-2110.9	568.6	853.6
5	74	584.1	949	-2173.6	732.1	1115.6
6	95	570.9	928	-2167.0	760.9	1253.2

The results of the selected model are presented in Table 3. The top section of the table reported the prevalence of each latent status at the baseline and after one year. 77.1% and 69.1% of students were in the risk-free status at baseline and the follow-up periods, respectively. Moreover, 2.3% and 5.1% of the students were in the multiple risk-taker status in the first and second time periods, correspondingly.

The second section of Table 3 shows the item-response probabilities, constrained to be equal at the two time periods and which form the basis for interpretation and labeling of the latent statuses. All of the statuses could be interpreted with respect to item-response probabilities. For example, the probabilities of being at the multiple risk-taker status were 68% for those who smoked cigarette, 48.6% for those who smoked hookah, 100% for those who used alcohol in the past 30 d, 22.4% for those who substance abused ever and 55.9% for those who had risky sexual behaviors.

The last part of Table 3 presents the transition probability matrix. This matrix expresses that 4.2% of students in risk-free status transited to tobacco user status, whereas 6.1% of transited to sexual risk-taker status. After one year, 22.4% of students in the tobacco user status transited to multiple risk-taker status.

**Table 3:** Results of the LTA model with four statuses and constrained item-response probabilities in two times

**Status**	**Risk-free**	**Tobacco user**	**Sexual risk-taker**	**Multiple risk-tacker**
**Prevalence of status**				
Time 1	0.771	0.123	0.083	0.023
Time 2	0.691	0.128	0.130	0.051
**Indicators (Risky Behaviors)**	**The probability of a “yes” response**			
Cigarette smoking	0.000	0.430	0.135	0.680
Hookah smoking	0.000	0.308	0.107	0.486
Alcohol use	0.003	0.000	0.008	1.000
Substance abuse	0.003	0.121	0.001	0.224
Risky sexual behaviors	0.000	0.057	1.000	0.559
Probability of transition			
**Latent status in time 1**	**Latent status in time 2**			
Risk- free	0.897	0.042	0.061	0.000
Tobacco user	0.000	0.776	0.000	0.224
Sexual risk-taker	0.000	0.000	1.000	0.000
Multiple risk-taker	0.000	0.000	0.000	1.000

## Discussion

This study is the first longitudinal study investigating the transition in risky behaviors and co-occurrence of risky behaviors among Iranian freshmen college students. The prevalence of cigarette and hookah smoking, alcohol consumption, substance abuse and risky sexual activities when they enter the university were 7%, 5.9%, 2.5%, 2.1% and 10%, respectively. These prevalences of risky behaviors were relatively less among the freshmen university students as compared to those reported in the general university and high school students^4, 10, 17, 18^. Previous studies on high school students have shown that risky behaviors such as tobacco smoking, alcohol consumption and illicit drug abuse were significantly associated with previous year average grades ^17, 18^. As the average high school GPA (grade point average) is an influential factor in the university admissions in Iran, it is expected to see young adults with less risky behaviors are more likely to get admitted to the universities in Iran. This might be the reason for lower the prevalence of risky behaviors in freshman university students than those in high school students. The prevalence of risk-taking behaviors among those early-adults who did not enter universities remained unknown in Iran. In the United States, a higher prevalence of smoking cigarette and marijuana in non-college young-adults, compared to their college counterparts, is reported^19^.

Although the prevalence of risky behaviors at the time of entrance to the university was low, the prevalence of cigarette smoking, hookah smoking, alcohol consumption, illicit drug abuse and sexual risky behavior increased by 4.7%, 1.6%, 2.8%, 0.9% and 6.4%, respectively, over the one year follow-up period. These results are consistent with other studies that demonstrated the elevating prevalence of risk-taking behaviors during the first year of college. The sense of independence, lack of parent’s monitoring, peer pressure and stressful life in the first year of the college have been reported as main factors with increased prevalence of risky behaviors^20, 21^. These results highlighted the importance of health education for this particular group of the population.

The results also showed that 89.7% of students in the risk-free status remained at this status at the second time-point assessment, while 4.2% of students in this status transitioned to tobacco smoker group and 6.1% transitioned to sexual risk-taker group. Higher prevalence of risky sexual behavior among freshmen (10%) compared to other risk behaviors, as well as the higher transition of students in risk-free group to sexual risk-taker group (6.1%) as compared to transition to tobacco smoker group (4.2%) is one of the noteworthy results of the current study. The latter findings maybe due to insufficient pre-college and college education about healthy sexual behaviors.

Sexual behaviors and risky sexual activities among early adults and university students in Iran are not well-studied and there is little available knowledge about their sexual pattern. The prevalence of risky sexual behaviors could be higher than what we reported in this study due to the religious and cultural constraints about premarital sexual activities. There is some evidence that warrants further attention to risky sexual behaviors among Iranian adults.

Our findings also indicated that the prevalence of multiple risk-taker students (i.e., those who have multiple risky behaviors) increased by 2.8% during freshman year. Additionally, 8% of students categorized in risk-free class at the beginning of the study involved in some type of risky behaviors during the study period. A study of first-year university students in Germany^22^ showed a high prevalence of multiple risk behaviors (18%). While the German study considered the lack of physical activity and unhealthy dietary habits as risky behaviors, the prevalence of smoking and alcohol drinking was found to be high.

Furthermore, the results of our study indicated that 22.4% of tobacco users transited to multiple risk-taker group during the first year of college. This means that tobacco smoking might predispose the initiation of other risky behaviors during the first year of college. In other words, tobacco smoking might act as a “gateway” in acquiring other risky behaviors such as illicit drug use, alcohol consumption and risky sexual behavior. Tobacco smoking among college students also predisposes co-occurrence of risky behaviors and progress towards multiple risk-taker group. The concept of tobacco smoking as a gateway for other risky behaviors has been supported by previous studies^20, 21^.

Several aspects of this study can limit the application of the findings. First, self-reported data used were subjected to inaccurate reporting of behaviors. Second, we lost a quarter of the participants in the follow-up assessment. This attrition may be associated with risk-taking behaviors; thus, there is a possibility of selection bias.

## Conclusion

The prevalence of risky behaviors increased during the freshman year. Tobacco smoking was found to predispose acquiring other risky behaviors as well as transferring to multiple risk-taker group. If we prevent smoking initiation during the first year of college, we might prevent acquiring other risky behaviors among college students.

## Acknowledgements

The authors would like to greatly acknowledge financial support for this study from Research Center of Psychiatry and Behavioral Sciences, Tabriz University of Medical Sciences. The authors also wish to thank all the participants of this study for their valuable cooperation and participation.

## Conflict of interest

The authors declare that there is no conflict of interests.

## Funding

This study was funded by Research Center of Psychiatry and Behavioral Sciences, Tabriz University of Medical Sciences.

## Highlights

- During the first year of college, risky behaviors became more common.
- Transition to tobacco smokers and sexual risk-taker groups were higher in freshman students.
- Tobacco smoking initiation may predispose other risky behaviors and transferring to the multiple high-risk groups.
